# Differences in Retinal Structure and Function between Aging Male and Female Sprague-Dawley Rats are Strongly Influenced by the Estrus Cycle

**DOI:** 10.1371/journal.pone.0136056

**Published:** 2015-08-28

**Authors:** Samaneh Chaychi, Anna Polosa, Pierre Lachapelle

**Affiliations:** 1 Department of Neurology and Neurosurgery, McGill University, Montreal, Quebec, Canada; 2 Department of Ophthalmology, Research Institute of the McGill University Health Centre, Montreal, Quebec, Canada; Dalhousie University, CANADA

## Abstract

**Purpose:**

Biological sex and age are considered as two important factors that may influence the function and structure of the retina, an effect that might be governed by sexual hormones such as estrogen. The purpose of this study was to delineate the influence that biological sex and age exert on the retinal function and structure of rodents and also clarify the effect that the estrus cycle might exert on the retinal function of female rats.

**Method:**

The retinal function of 50 normal male and female albino Sprague-Dawley (SD) rats was investigated with the electroretinogram (ERG) at postnatal day (P) 30, 60, 100, 200, and 300 (n = 5–6 male and female rats/age). Following the ERG recording sessions, retinal histology was performed in both sexes. In parallel, the retinal function of premenopausal and menopausal female rats aged P540 were also compared.

**Results:**

Sex and age-related changes in retinal structure and function were observed in our animal model. However, irrespective of age, no significant difference was observed in ERG and retinal histology obtained from male and female rats. Notwithstanding the above we did however notice that between P60 and P200 there was a gradual increase in ERG amplitudes of female rats compared to males. Furthermore, the ERG of premenopausal female rats aged 18 months old (P540) was larger compared to age-matched menopausal female rats as well as that of male rats.

**Conclusion:**

Our results showed that biological sex and age can influence the retinal function and structure of albino SD rats. Furthermore, we showed that cycled female rats have better retinal function compared to the menopausal female rats suggesting a beneficial effect of the estrus cycle on the retinal function.

## Introduction

Age and biological sex are two of the most important regulator of our day-to-day body functions, including retinal function. The influence of biological sex on the human retinal function, as determined with the electroretinogram (ERG), has been known for more than 60 years [1]. Electroretinograms are usually reported to be of larger amplitudes in women compared to men [2]. In a recent study, the amplitudes of scotopic and photopic ERGs of female subjects were reported to be on average 29% larger than their male counterparts [3]. From the structural point of view, spectral domain optical coherence tomography (SD-OCT) did reveal sex-related differences in the mean thickness of the retinal layers at the macula. It showed that the Outer Nuclear Layer (ONL), the Outer Plexiform Layer (OPL) and the Inner Nuclear Layer (INL) were thicker in men, while the Nerve Fiber Layer (NFL) was thicker in women [4]. It is thought that sex-related functional and structural differences in body tissues, including the retina, might be governed by the remarkable male-female differences in sex hormone profiles. Of interest, the presence of estrogen receptors in different retinal layers [5,6] suggests that this sexual hormone might play an important role in the normal functioning of this tissue [7,8]. Furthermore, a possible modulatory effect of the menstrual cycle and the accompanying hormonal fluctuations, especially estrogen, was observed on several ocular structures, including the retina [9,10].

The effect of age on retinal function and structure has also been demonstrated in human and animals [11,12,13]. Aging is one of the most important contributors to cumulative oxidative stress that could result in the gradual deterioration in function and structure of different tissues including the retina [13,14,15]. It has been reported that the antioxidant properties of some tissues such as the heart, kidneys, liver and brain was significantly higher in female rats explaining the longer lifespan in females [16]. Given that estrogen has anti-oxidant effects on different tissues of the body and exerts neuroprotection without having to involve a receptor-mediated process [17,18], it could be proposed that sex might also influence the normal aging process. Although the plasmatic levels of estrogen in men remain relatively constant throughout life, in women it fluctuates over a larger range [19]. At menopause, the ovaries stop producing sex hormones, while in men the testes never stop producing testosterone, which is partly converted into estradiol in the neural tissues [19]. It may explain the higher incidence of some age-related ocular pathologies such as cataract [20], glaucoma [21] and acquired macular degenerations [22] in postmenopausal women that most probably result from the sudden decline in circulating estrogen. Supporting the latter claim, previous studies reported a reduced occurrence of cataract and age-related macular degeneration in women on estrogen replacement therapy compared to those not receiving hormonal supplementation [22,23,24,25].

In view of the above, the purpose of the present study was to investigate the effect of biological sex and age on the retinal function and structure of aging male and female albino Sprague-Dawley (SD) rats. Our results showed that in female rats there was an increase in ERG response amplitudes noticed between 2 and 6.5 months of age while in age-matched male rats the ERG amplitudes remained stable during the same period. The latter results would suggest a beneficial effect of the estrus cycle on the retinal function. Furthermore, our results also showed that premenopausal female rats had larger ERG amplitudes compared to menopausal rats, further confirming the role of the estrus cycle on the retinal function.

## Methods

### Animals

All experiments were conducted in accordance with the Association for Research in Vision and Ophthalmology (ARVO) statement for the use of animals in ophthalmic and vision research and were approved by the McGill University-Montreal Children's Hospital animal care committee. Adult female SD rats (Charles River Laboratories, St-Constant, QC) were ordered at 15 days of gestation and kept in the normal environment of animal facility where they were allowed food and water *ad libitum*. Rat pups remained with their mom until weaning at P21 when they were separated according to their sex.

### Experimental groups

The retinal function of 50 normal male and female SD rats was investigated with the ERG at age P30, P60, P100, P200 and P300 [5–6 rats per sex and age groups] in order to evaluate the effect of sex and age on the retinal function. The retinal samples were collected right after each recording session at P30, P60, P100 and P300 [2–4 rats per sex at each age] to assess the role of sex and age on the retinal structure.

In parallel, the ERGs of 6 normal female SD rats were obtained at 18 months (P540). Given that the menopausal age of laboratory rats is reported to be between 15 and 18 month [26], vaginal smears (to determine the stage of the estrous cycle) were taken from the female rats before the ERG recordings in order to study the effect of estrus cycle (equivalent to menstrual cycle in women) on the retinal function. Results were compared to those obtained from 5 male SD rats aged 18 months.

### Vaginal smear sampling

Use of vaginal smear (VS) technique, allows one to identify the different estrus cycle phases (proestrus, estrus, metestrus and diestrus) based on the proportion among three types of cells: nucleated epithelial cells, cornified cells and leukocytes, the proportion of which varies according to the plasmatic level of estrogen [27].

In this study the VS technique was used in order to determine the menstrual status in female rats through observation of different cell types in the vaginal secretions. The six 18 month-old female rats were housed in standard cages, 2 per cage, in the animal care facility. They were kept in a controlled temperature room (22°C), with a 12hr light:12hr dark cycle. Since the duration of the estrus cycle in rodents lasts 4–5 days, vaginal secretions were collected over five consecutive days between 10 and 11 AM with a plastic pipette filled with 10μL of normal saline (NaCl 0.9%) that was inserted into the vagina, but not deeply [27]. The saline was then flushed into the vagina and sucked back into the pipette. The resulting fluid was placed on glass slides, stained with 0.1% toluidine blue, following which pictures were taken with a Zeiss microscope (Zeiss Axiophot, Zeiss microscope, Germany: 40X) equipped with a digital camera.

### Electroretinography

ERGs were recorded with a data acquisition system (Acqknowledge; Biopac MP100; Biopac System Inc., Goleta, CA, USA) as previously described [28,29,30]. Briefly, the rats were kept in the dark for at least 12hr (i.e. overnight) before recording in order to enhance retinal sensitivity [31]. The experimental procedure was done under a dim-red light. First, the animals were anaesthetized with an intramuscular injection of ketamine (85mg/kg) and xylazine, (5mg/kg). The pupils were dilated with one drop of 1% Mydriacyl, and the cornea was anesthetized with a drop of 0.5% Alcaine. The animals were laid on their right side in a recording box [32] in which a rod desensitizing background light and a photo-stimulator had been installed (model PS 22, Grass Instrument, Quincy, MA). The retinal potential was captured at the cornea with a DTL fiber electrode (27/7 X-Static silver coated conductive nylon yarn: Sauquoit Industries, Scranton, PA, USA) acting as the active electrode [33]. It was maintained on the cornea with an ophthalmic liquid gel (Tear-Gel; Novartis Ophthalmic, Novartis Pharmaceuticals Inc., Canada), which was also used to prevent corneal dryness. Reference (E5 disc electrode; Grass Technologies, Quincy, MA, USA) and ground (E2 sub-dermal electrode; Grass Technologies, Quincy, MA, USA) electrodes were placed in the mouth and the tail, respectively. Twenty flashes of white light (flash duration: 20μ sec; inter-stimulus interval: 10sec) at increasing intensities, starting from the lowest (- 6.3 log_**·**_cd_**·**_s_**·**_m^-2^) to the brightest (0.9 log_**·**_cd_**·**_s_**·**_m^-2^) were delivered to produce the scotopic ERGs luminance-response function. Following the scotopic ERG recordings, a background light of 30 cd.m^-2^ was opened and, after 20min of light adaptation, the photopic ERGs were recorded in response to a flash of 0.9 log_**·**_cd_**·**_s_**·**_m^-2^ (average of 20 flashes with inter-stimulus interval 1sec) in intensity.

### Retinal histology

Following each recording session (P30, P60, P100 and P300) two to four male and female rats (per age groups) were euthanized with CO_2_ asphyxiation and their retinas were collected for histology. Briefly, an orientation suture was placed on the nasal conjunctiva of both eyes then the eyes were enucleated and immersed for 3 hours in 3.5% glutaraldehyde for fixation. After the removal of the cornea and lens, the eyecups were placed in 3.5% glutaraldehyde overnight. On the following day, the eyes were immersed in a solution of 1% osmium tetroxide (OsO_4_) for 3 hours. Then, the eyes were sequentially dehydrated with 50, 85, 90, 95 and 100% ethanol and propylene oxide, respectively. Finally, the samples were embedded in resin (Durcupan ACM Fluka epoxy resin kit, Sigma-Aldrich, Canada) and kept in an oven at 55–60°C for 48 hours. The samples were cut along the vertical meridian of the eye passing through the optic nerve head (ONH) (Leica EM UC6 microtome, Leica microsystem, USA) in ultra-thin sections of 1.0 μm and stained with 0.1% toluidine blue. Retinal pictures were taken with a Zeiss microscope (Zeiss Axiophot, Zeiss microscope, Germany: 40X) equipped with a digital camera.

For each rat, the thickness of the entire retina as well as that of each retinal layer (i.e., RPE, OS/IS, ONL, OPL, INL, IPL, RGCL/FL) were measured on 6 different histological sections (taken at every 680 μm interval from the ONH) taken from the superior retina using the AxioVision software (version 4.8.2.0; Carl Zeiss Microscopy GmbH, Jena, Germany). On each section thickness measurement was done at two to three randomly selected locations.

### Data Analysis

ERG analysis was performed according to the standard practice [34] as previously reported by us [29,30,35]. The amplitude of the a-wave was measured from baseline to the most negative trough, while the amplitude of the b-wave was measured from the trough of the a-wave to the most positive peak of the retinal response. The maximal rod-mediated b-wave (rod V_max_), representing the maximal function of the rods without the contribution of cones, was defined as the first ERG response (of the scotopic luminance response function) where a measurable a-wave was obtained. The mixed rod-cone a-wave and mixed rod-cone b-wave refer to scotopic ERGs evoked to the highest flash intensity available (0.9 log cd.sec.m^-2^). Finally, in order to investigate the possible age-related changes in the retinal function over time, a maturation index of ERG parameters was calculated. This index represents the ratio between ERG measurements obtained at P60, P100, P200, P300 and those at P30 (i.e. P60/P30 x 100). All values are represented as mean ± 1 standard deviation (SD). Two-way ANOVA followed by a post-hoc Tukey or Bonferroni test (for significant ANOVA results) was performed to evaluate the retinal function and structure of male and female rats at different ages. A P < 0.05 was considered as statistically significant. All statistical analyses were performed with GraphPad Prism (GraphPad Software Inc., San Diego, CA, USA).

## Results

### The effects of biological sex and age on retinal function

**Fig 1:**
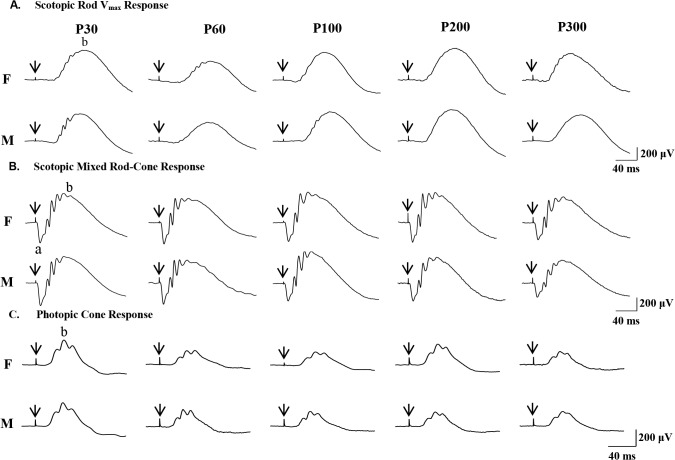
Representative scotopic rod V_max_ (A) and mixed rod-cone (B) ERGs and photopic ERG (C) recorded from a male and a female rat aged 30, 60, 100, 200 and 300 days old (age identified as P at the top up each column). Vertical arrows correspond to the stimulus onset. Horizontal calibration: 40 msec. Vertical calibration: 200 μV. Abbreviations: a: a-wave; b: b-wave. F: female rat. M: male rat.

Fig 1 shows representative scotopic rod V_max_ responses (A), scotopic mixed rod-cone responses (B) and photopic cone responses (C) obtained from male and female rats at age P30, P60, P100, P200 and P300 (from left to right). Amplitude measurements of ERG parameters obtained from male and female rats at five different ages are reported in Table 1. Irrespective of age, no significant differences in amplitude and morphology were observed in ERG responses between males and females (P > 0.05). In both sexes, the scotopic and photopic ERGs were significantly attenuated at P60 compared to those at P30 (P < 0.05), whereas the amplitude drop measured for the rod V_max_ at P60 was not remarkable (Table 1).

**Table 1:** Group data reporting the average amplitudes (in μV) of the scotopic ERGs, the photopic b-wave and rod V_max_ in male and female rats obtained at 30, 60, 100, 200 and 300 days of age. Values are given as mean ± SD.

Parameter	Gender	Age (Days)				
	M/F	30	60	100	200	300
**Scotopic a-wave ± SD (**μ**V)**	**F**	440.2 ± 122.3	307.0 ± 36.2**	322.3 ± 55.8*	343.8 ± 67.4	283.6 ± 16.6**
	**M**	489.0 ± 52.1	324.9 ± 33.4***	304.3 ± 37.4***	294.5 ± 74.0***	266.9 ± 11.6***
**Scotopic b-wave ± SD (**μ**V)**	**F**	971.9 ± 274.7	691.9 ± 74.5*	744.0 ± 61.5	827.3 ± 177.8	706.9 ± 57.9*
	**M**	1075.5±111.8	765.0 ± 128.2**	831.2 ± 126.6	735.3 ± 144.2**	707.2 ± 32.7**
**Photopic b-wave ± SD (**μ**V)**	**F**	254.0 ± 45.6	170.9 ± 23.9***	165.9 ± 21.0***	195.7 ± 32.4*	182.4 ± 38.1**
	**M**	225.2 ± 52.3	163.7 ± 35.9*	190.4 ± 31.7	174.5 ± 10.8	171.4 ± 10.4
**Rod Vmax ± SD (**μ**V)**	**F**	550.6 ± 131.1	422.3 ± 55.7	456.1 ± 126.3	575.3 ± 153.6	481.8 ± 36.4
	**M**	574.5 ± 41.2	402.9 ± 36.9	419.6 ± 202.3	486.9 ± 85.4	460.9 ± 59.1

* *p* < 0.05, ** *p* < 0.01 and *** *p* < 0.001 indicate statistical difference between the amplitudes recorded at the specified age and those at P30.

**Fig 2:**
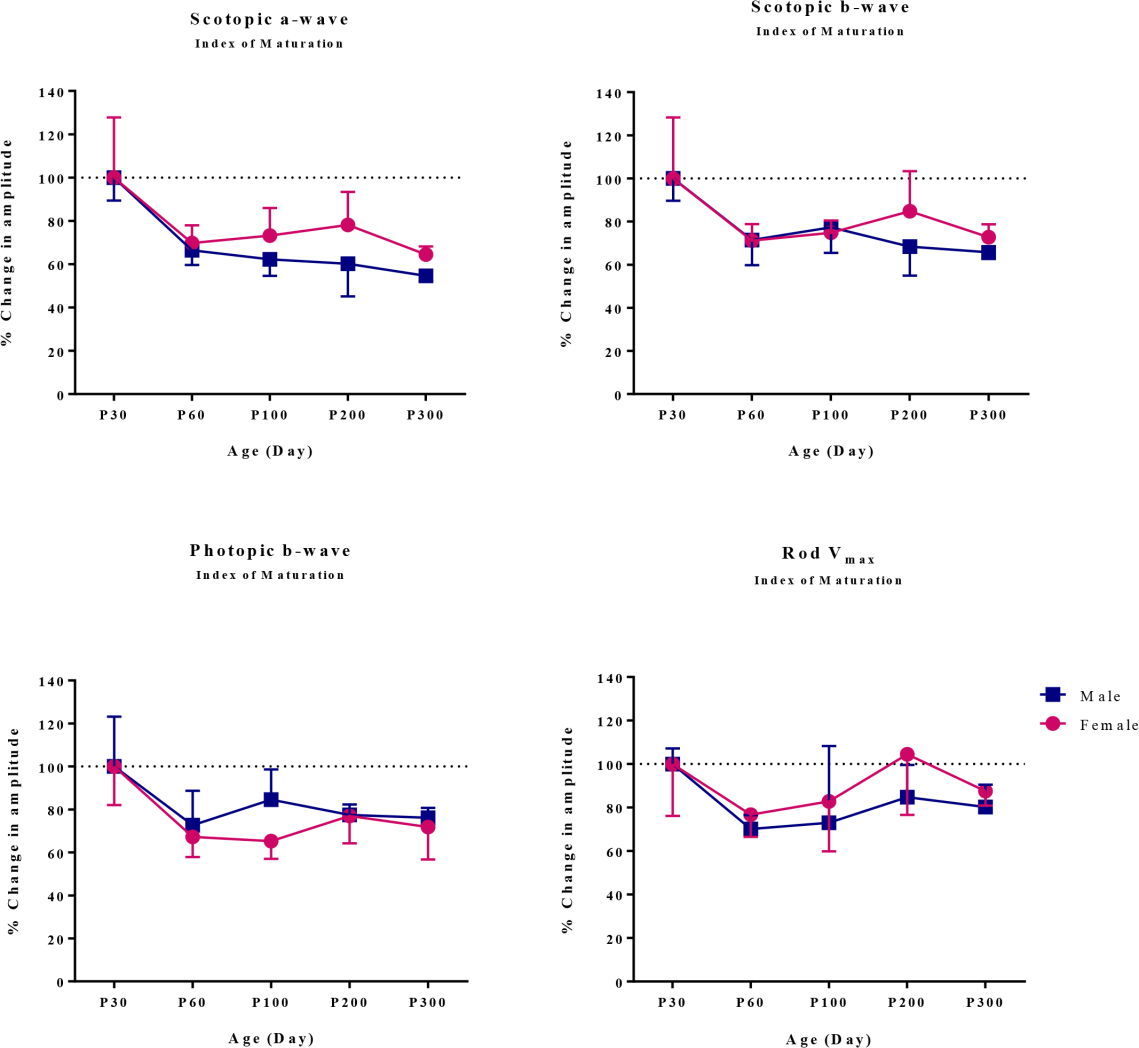
Maturation of retinal function in male and female rats (expressed of P30 value in %) at 30, 60, 100, 200 and 300 days of age. Data was normalized to P30 values. Values are given as mean ± SD.

**Fig 3:**
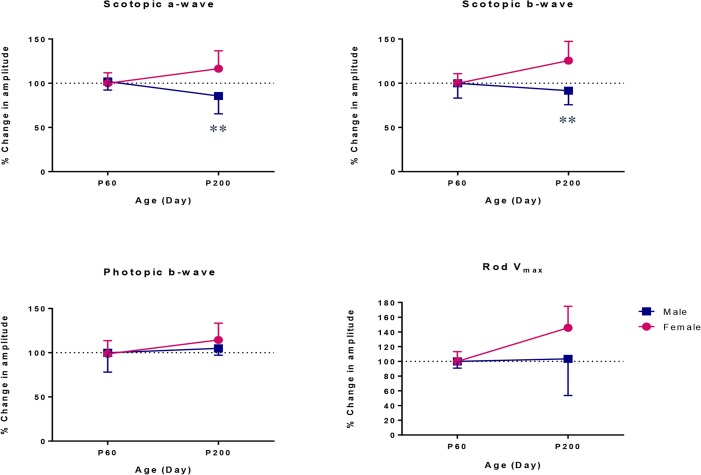
Percent changes in retinal function of male and female rats between P60 and P200 as determined with the retinal maturation index. The retinal maturation index represents the average (percentage) of the ratios (P200/P60 X 100) for ERG parameters (scotopic a-wave, scotopic b-wave, photopic b-wave, rod V_max_). ** P < 0.01 indicates significant difference between male and female rats at P200. Values are given as mean ± SD.

Fig 2 shows the maturation index of the retinal function in male and female rats. Interestingly, the maturation indexes for ERG parameters in female rats gradually increased between P60 and P200, while those of males did not show this trend. This is better exemplified at Fig 3 where the ERG responses of male and female rats measured at P200 were normalized to those obtained at P60. As shown at Fig 3, ERG responses in females increased noticeably between P60 and P200, while those of males decreased except for the cone-mediated ERG and rod V_max_ that showed small increases (Fig 3C and 3D). At P200, the growth in amplitude of the rod-cone mediated ERG was significantly lower in male compared to female rats [scotopic a-wave: females, 116.41% vs. males, 85.55% (P < 0.01); scotopic b-wave: females, 125.58% vs. males, 91.58% (P < 0.01)] (Fig 3A and 3B).

Afterwards, the retinal function in male and female rats decreased from P200 to P300. At P300, rod-cone mediated ERGs (scotopic ERGs) obtained from both sexes and the photopic ERGs of female rats were significantly attenuated compared to those obtained at P30 (P < 0.05) (Table 1).

### The effect of estrus cycle on retinal function

**Fig 4:**
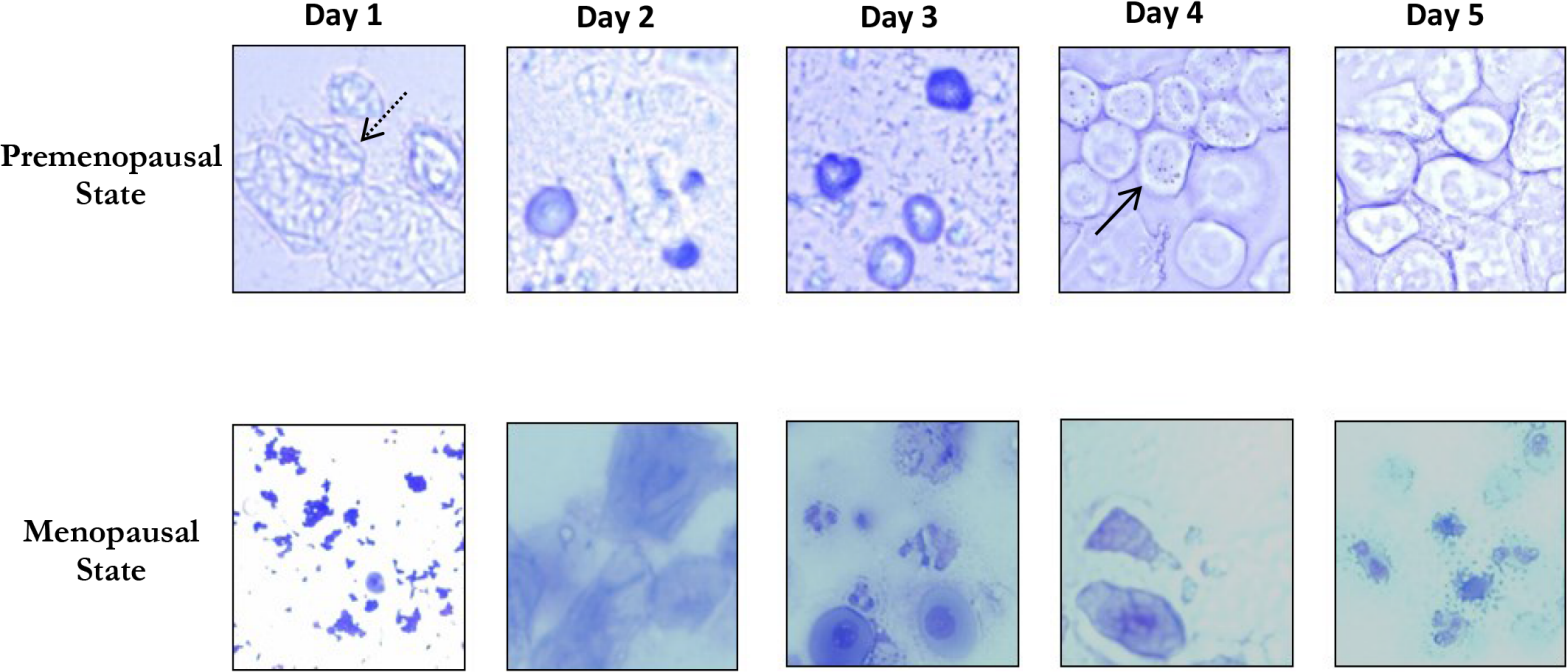
Representative examples of vaginal smears obtained from premenopausal (the upper images) and menopausal (the bottom images) female rats aged 18 months collected over five consecutive days. A typical estrus cycle (with the four consecutive phases: proestrus, estrus, metestrus, and diestrus) could be identified in premenopausal rats. Proestrus phase was identified with the presence of nucleated epithelial cells (solid line in image taken at day 4); Estrous phase was recognized with the presence of cornified epithelial cells (dashed line in image taken at day 1); the other two phases (Metestrus and Diestrus) were mainly distinguished with the leukocyte infiltration (images taken at day 2 and 3). No equivalent distinct estrus phase could be observed in menopausal group. The images were taken with a 40X objective.

**Fig 5:**
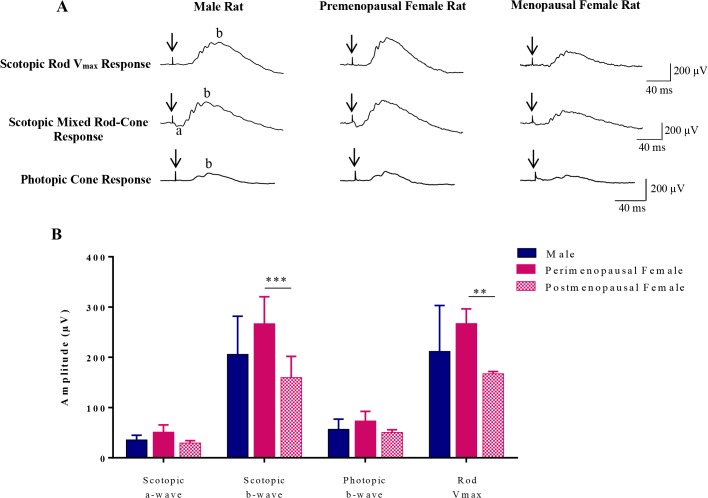
(A) Representative scotopic (mixed rod-cone and rod V_max_) and photopic ERGs recorded from a male rat (first column), premenopausal and menopausal female rats (second and third column, respectively) at age 18 months. (B) Comparison of ERG amplitudes (scotopic a-wave, scotopic b-wave, photopic b-wave, and rod V_max_) measured from male rats, premenopausal and menopausal female rats. **P < 0.01; *** P < 0.001 indicate significant differences between premenopausal and menopausal female rats. Values are given as mean ± SD. Vertical arrows correspond to the stimulus onset. Horizontal calibration: 40 msec. Vertical calibration: 200 μV. Abbreviations: a: a-wave; b: b-wave.

Six, 18 month-old, female rats were categorized into premenopausal (N = 2) and menopausal (N = 4) groups according to their vaginal smears. Fig 4 shows representative vaginal smears of both groups. Estrus cycle phases were identified in premenopausal female rats, whereas no distinct phase could be observed in menopausal female rats. Representative scotopic and photopic ERG and rod V_max_ obtained from premenopausal and menopausal groups as well as from age-matched males are presented at Fig 5A. Group data are shown in Table 2.

**Table 2:** Group data reporting the average amplitudes (in μV) of the mixed rod-cone a-wave (scotopic a-wave), mixed rod-cone b-wave (scotopic b-wave), photopic cone b-wave, and rod V_max_ measured for male rats, premenopausal and menopausal female rats at age of 18 months. Values are given as mean ± SD.

	No. of Animals	Mixed Rod-Cone a-wave	Mixed Rod-Cone b-wave	Photopic Cone b-wave	Rod V_max_
**Male**	**N = 5**	35.5 ± 9.4	205.8 ± 76.0	56.6 ± 20.2	211.5 ± 91.5
**Premenopausal female**	**N = 2**	51.1 ± 14.2	266.7 ± 53.9	73.3 ± 18.9	267.1 ± 29.1
**Menopausal female**	**N = 4**	29.4 ± 4.7	159.8 ± 41.9***	50.6 ± 5.2	167.3 ± 4.4**

** *p* < 0.01 and *** *P* <0.001 indicate statistically difference between premenopausal and menopausal female rats.

Premenopausal females showed higher ERG responses compared to the menopausal females. The mixed rod-cone b-wave (scotopic b-wave) and rod V_max_ were significantly attenuated in the menopausal group compared to the premenopausal (P = 0.0009 and P = 0.002, respectively) (Fig 5B). Interestingly, the retinal function of premenopausal females was better preserved compared to aged-matched males, while ERG responses in menopausal female rats were of lower amplitude compared to those of age-matched males, suggesting an effect of the estrus cycle on the retinal function of female rats.

### The effects of biological sex and age on retinal structure

**Fig 6:**
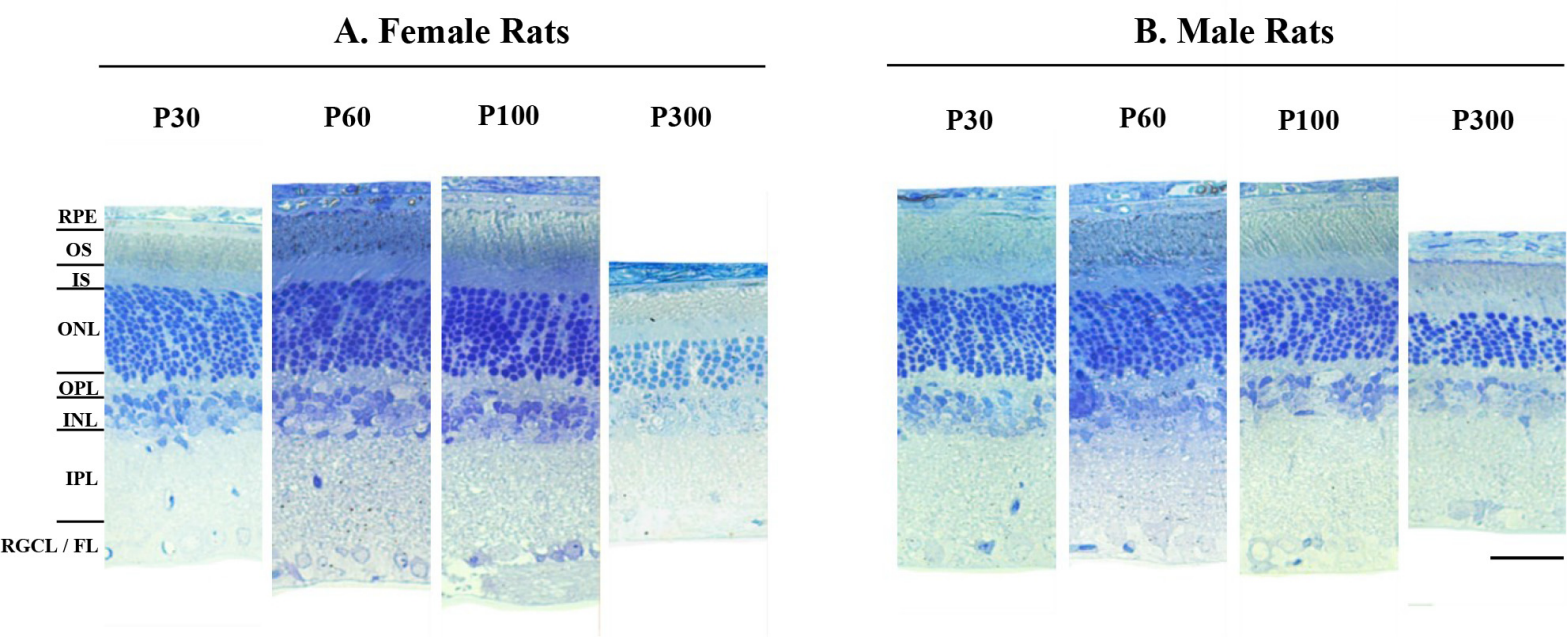
Representative retinal sections obtained at 30, 60, 100, and 300 days of age from female (A) and male adult SD rats (B). Images were taken between 1020 μm and 1700 μm from the optic nerve head in the superior retina. Abbreviations: RPE: retinal pigmented epithelium; OS: outer segment; IS: inner segment; ONL: outer nuclear layer; OPL: outer plexiform layer; INL: inner nuclear layer; IPL: inner plexiform layer; RGCL/FL: retinal ganglion cell layer/fiber layer. Calibration bar: 75μm.

The maturation-induced changes in retinal function described earlier were also accompanied with changes in retinal structure. Representative retinal cross sections obtained at postnatal days 30, 60, 100, and 300 from male and female rats are shown in Fig 6. In both sexes the retinal thickness decreased with age. At P300, the retina of female rats was 20.14% thinner than P30 measures compared to 10.06% for males (P > 0.05).

**Fig 7:**
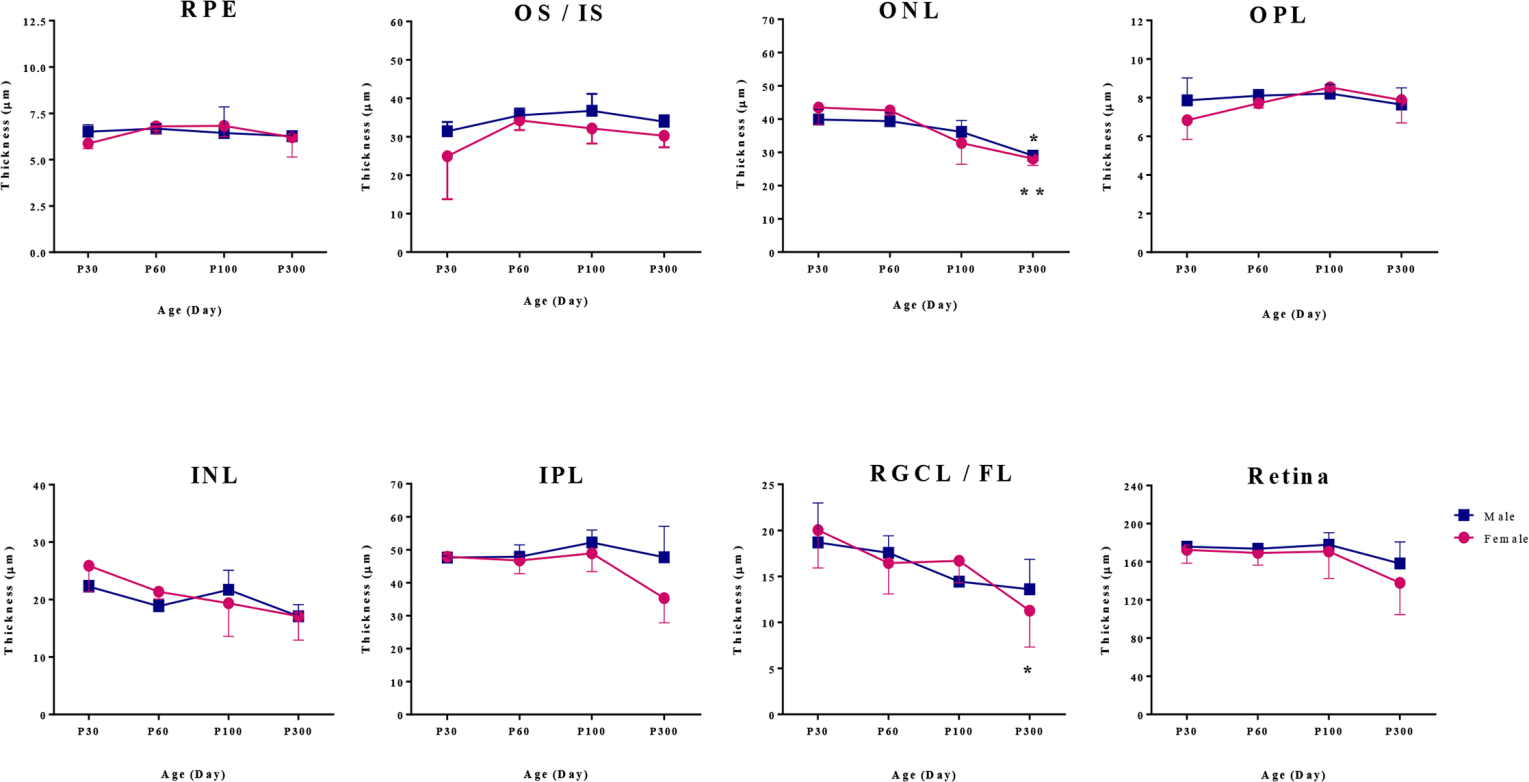
Quantitative assessment of retinal structure in male and female rats. Retinal layer thicknesses in female and male rats (N = 2-4/age) were measured at 30, 60, 100, and 300 days of age. * P < 0.05; *** P < 0.001 indicate significant differences between the thicknesses measured at P300 and those at P30. Values are given as mean ± SD.

As shown at Fig 7, the quantitative assessment of the different retinal layers did not evidence any significant difference in retinal layer thicknesses between male and female rats, irrespective of age. Interestingly, the ONL thickness in adult male and female rats aged P300 was significantly thinner than that measured at P30 [ONL thickness in male rats at P300 vs. P30: 29.003 ± 0.67 μm vs. 39.84 ± 3.07 μm (P = 0.01); ONL thickness in female rats at P300 vs. P30: 28.08 ± 1.98 μm vs. 43.48 ± 5.15 μm (P = 0.001)], suggesting that the photoreceptors were more vulnerable to the aging process. Moreover, of all the inner retinal layers, the RGCL/FL was that most affected by the aging process in females [RGCL/FL thickness in female rats at P300 vs. P30: 11.27 ± 3.9 vs. 20.02 ± 4.1 (P = 0.02)].

It is noteworthy that alterations in the thicknesses of the outer retinal layers (RPE, OS/IS, ONL and OPL) and INL showed nearly similar patterns in male and female rats over time. However, during the P100 to P300 period, the thicknesses of the IPL and RGCL/FL decreased more in female compared to male rats [IPL thickness at P300 vs. P100: male, 47.73 ± 9.4 μm vs. 52.21 ± 3.8 μm (8.59% decrease); female, 35.31 ± 7.4 μm vs. 48.92 ± 5.5 μm (27.83% decrease). RGCL thickness at P300 vs. P100: male, 13.60 ± 3.2 μm vs. 14.42 ± 0.4 μm (5.69% decrease); female, 11.27 ± 3.9 μm vs. 16.69 ± 2.3 μm (32.49% decrease)]. (Fig 7)

## Discussion

To our knowledge, this is the first study to explore sex-related differences in retinal function and structure during the normal aging process of albino SD rats. In this study, we showed that the retinal function of female rats was better preserved compared to age-matched male rats, particularly between ages P60 and P200. We believe that this difference might be explained by the positive effect of the estrus cycle on the retinal function, a claim also supported with our result showing ERG responses of larger amplitudes in premenopausal female rats compared to menopausal rats.

### Age and sex-related changes in retinal function

In this study, we show that the normal aging process modulates the retinal function in both male and female SD rats, as shown with the attenuation of ERG amplitudes measured between P30 and P300 (Fig 2). Age-dependent alterations in ERG responses have been extensively investigated in both human and rat [2,11,36]. In human studies, it has been reported that the amplitudes of the ERG a- and b-waves decline with normal aging [2], indicating age-dependent alterations in the outer and inner retinal function, respectively. However, the study of Freund et al. [12] on healthy subjects showed that despite a significant decrease in the amplitude of the dark adapted a-wave, the amplitude of the dark adapted b-wave did not decline with age resulting in an increased b/a wave ratio, indicating more vulnerability of the outer retinal function to the normal aging process. Several factors, including subtle changes in ocular media and/or reductions in photopigment contents [2], or a gradual loss of retinal cells such as photoreceptor, bipolar or Müller cells in the aging retina [37,38], have been proposed as the main reasons for the age-dependent attenuation in ERG amplitudes.

Regardless of age, we did not observe any significant male-female differences in the electrical responses of the retina when female ERGs are compared with control age-matched male ERGs. This finding is in agreement with a previous study which concluded that the photopic flash ERG obtained from male and female SD rats aged 9 to 12 weeks were identical [39]. However, while in our study, the ERG of male rats tended to remain stable between P60 and P200, in female rats there was an increase in all ERG parameters (Fig 3). Of interest, this age episode (P60-P200) also corresponds to that where the frequency of estrus cycle starts to increase to reach maximum value at age P240 [40], suggesting that the estrus cycle and accompanying circulating hormones, especially estrogen, might influence the retinal function in female rats. Given that the retina is a highly vascularized tissue, the discovery of estrogen receptors located in the different retinal layers [5,6] also supports the claim that the circulating estrogen may influence the retina. Moreover, it has been shown that the level of antioxidant enzymes such as catalase and superoxide dismutase (SOD) were remarkably increased in retinal tissues obtained from 6 months old albino Wistar rats [13] that is at an age where the estrus cycle frequency is nearly maximal [40]. Of interest, one study reported that estrogen enhanced the expression as well as the activity of extracellular SOD (ecSOD) and mitochondrial manganese SOD (mnSOD) by upregulating the transcriptional pathways [41]. Regarding the modulatory effects of estrogen on the SOD level, our demonstration of a better preservation of retinal function in female rats may have resulted from the gradual increase in the estrus cycle frequency and accompanying increase in the level of plasmatic estrogen, thus resulting in an increase in antioxidant activity within the retina, a tissue known to be highly susceptible to age-related oxidative damage [42]. Additionally, antioxidant properties of estrogen in neural tissue including the retina [43,44,45] boost the antioxidative effects of SOD leading to a better protection of the retinal function against the gradual deterioration of function due to age-associated oxidative insult.

The results obtained in the second part of our study further confirm the impact of the estrus cycle on the retinal function. As illustrated in this study (Fig 5), the ERG amplitudes were larger in premenopausal compared to menopausal female SD rats and also larger than those obtained from age-matched male rats. In contrast, following menopause, the ERG responses in female rats declined noticeably compared to age-matched male rats. These findings suggest a beneficial effect of the estrus cycle and its related hormonal changes upon the retinal function that may explain the higher prevalence of age-related retinal degeneration in women after menopause [22].

Interestingly, it appears that the rod-mediated ERG is more influenced by the estrus cycle and accompanying hormonal fluctuations compared to the cone-mediated ERG. As shown in this study (Fig 5B), in premenopausal rats the scotopic ERGs (rod V_max_ and mixed rod-cone responses) were significantly larger than those of menopausal rats, while the photopic ERG (representing cone function) did not show this remarkable discrepancy. This effect is also emphasized with the results at Fig 3 where we show that in female rats the changes in rod V_max_ amplitudes measured between P60 and P200 were larger than those measured for photopic b-wave, suggesting a more important influence of the estrus cycle on rod function. Furthermore, at age P200, the difference between male and female responses was larger for the scotopic rod V_max_ ERG compared to photopic ERG (Fig 3), suggesting better preserved rod function in female rats. The reason for this rod-cone discrepancy remains to be elucidated.

### Age and sex-related changes in retinal structure

Retinal histology obtained at P30, P60, P100 and P300 did not reveal significant differences in retinal layer thicknesses between male and female rats, irrespective of age. Interestingly, we found that the photoreceptor layer was the retinal structure most affected by the normal aging process of male and female rats. From a pathophysiological point of view, it is believed that age-associated photoreceptor loss might be attributable to age-dependent changes in the RPE [46,47] which has a crucial role in maintaining the cellular integrity of the photoreceptors [48]. Any deterioration of the RPE function could affect the survival of retinal cells, including the photoreceptors which are highly dependent upon the proper functioning of the RPE [49]. Additionally, it has been hypothesized that the photoreceptor loss due to aging could be related to the damage induced by excitatory amino acids such as glutamate [11]. In response to the light stimulus, the photoreceptors release glutamate [50] which is then captured by the Müller cells. Aging Müller cells may not be as efficient to catch the released glutamate, therefore augmenting the extracellular levels of glutamate and, consequently, leading to a further increase in photoreceptor cell death at long term [11]. As illustrated in our study, except for the photoreceptor layer, the other layers of the outer retina (i.e.: RPE, outer and inner segments of the photoreceptors and OPL) of male and female rats did not change over time.

Our results suggest that biological sex did not affect noticeably the age-related loss of photoreceptors since both sexes showed a nearly similar decrement in ONL thickness with age. This contrasts with DiLoreto et al.’s study [51] which showed that compared to male, the photoreceptor degeneration documented in female Fischer 344 (F344) rats was delayed and less severe. Interestingly, in their study, the higher rate of retinal degeneration in the male F344 rats was not observed in age-matched male SD rats. This finding suggests a strain difference in the severity of age-dependent retinal degeneration and may explain why in our study, we found an almost equal rate of degeneration in male and female SD rats.

It has been shown that the aging phenomenon is associated with the loss of neurons in the inner retina, particularly at the level of the ganglion cell and nerve fiber layers [52,53]. In one study conducted by Sandalon et al. [54], it was shown that the significant RGC loss was initiated between the age 4 and 12 months in rats, a finding in accord with the results presented herein. We found that the thinning of the inner retinal layers (INL, IPL, and RGC/FL) over time was more pronounced in female rats particularly after 3 months of age (P100). Moreover, the sex-related differences in IPL and RGCL/FL thicknesses between P100 and P300 appeared to be mostly responsible for the sex-related difference measured for the entire retina. As shown in the present study (Fig 7), the age-related deterioration of RGCL/FL was significantly more pronounced in 10 month-old female rats compared to 1-month-old female rats, a finding that might be attributable to the gradual decrease in estrus cycle frequency following its peak at 8 months of age [40]. In a recent study it was shown that the ganglion cells and nerve fiber layers suffered more from the normal aging process in female albino Wistar rats compared to their male counterparts [13]. Of interest, these results could be explained with the findings of Kobayashi et al. [5] who showed an enhanced expression of estrogen receptors in the inner retinal layers including the nerve fiber layer, the ganglion cell layer, and the inner nuclear layer of 8-week-old Lewis rats. Although, the latter study did not report significant sexual differences in the distribution of estrogen receptor protein in the rat retina, it could be that the responsiveness of estrogen receptor differs between male and female.

## Conclusion

In summary, our results demonstrate that biological sex and age can influence the retinal structure and function in albino SD rats. Of note, it seems that estrogen, the most important fluctuating hormone of the estrus cycle, modulates the retinal function of female rats. This claim is supported with the larger ERG amplitudes that we obtained from female rats, particularly between 2 and 6 month of age. In addition, this claim is further confirmed with our result showing ERG responses of larger amplitudes in premenopausal female rats compared to menopausal rats.
